# A Cool Detective

**Published:** 1885-11

**Authors:** 


### A Cool Detective.

Highwaymen in the mining states seldom operate upon a stage-coach with "U. S. M." on it. They know that these initlals stand for the United States Mail, and are a pledge that the whole power of the government will be used to capture them.

The detectives in the government service are quiet men, courteous in manner and gentle in speech. Mr. Hayes tells, in his book on "New Colorado," of one whom he met, who wore gold spectacles, and looked like a German professor. Yet this man alone took two mail robbers from the North of Texas. At one place their friends planned a rescue. He quietly informed his prisoners that, while their friends could undoubtedly kill him, they might be sure that the first motion would send both of them into eternity. Not a man in the crowd moved a finger.

On one occasion, a celebrated detective was on a stage which was attacked by two masked men. The first he knew was that two revolvers were thrust in the coache's window, with the command, "Hands up, gentlemen!"

The highwaymen "had the drop" on the passengers, which, in their vocabulary, meant the certainty of their being able to kill before being harmed themselves. To his disgust, the detective was compelled to give up his watch and money.

As the robbers left, he put his hand down in the "boot," and to his delight it touched a carbine. Asking the driver to go on a little further, and then stop and wait for him, he went back alone.

The two men, unsuspicious of danger, were "divvying up" the spoils in the middle of the road. This was just what the detective had calculated on.

"Now, you scoundrels, it's my turn," he shouted, covering them by the repeating carbine. "Throw up your hands or I'll shoot."

The robbers, at his command, stepped one side, holding up their hands, while he picked up their revolvers, It was not many minutes before the astonished passengers saw the highwaymen walking down the road, with the cool detective following. They were taken in the coach and finally lodged in jail.

The hero was Gen. Charles Adams, who subseqnently went alone among the Utes and secured the release of the women captives from the White River Agency.

